# The transcription factor *Krüppel homolog 1 *is linked to hormone mediated social organization in bees

**DOI:** 10.1186/1471-2148-10-120

**Published:** 2010-04-30

**Authors:** Hagai Shpigler, Harland M Patch, Mira Cohen, Yongliang Fan, Christina M Grozinger, Guy Bloch

**Affiliations:** 1 Department of Evolution, Systematics, and Ecology, The Alexander Silberman Institute of Life Sciences, Hebrew University of Jerusalem, Jerusalem, Israel; 2 Department of Entomology and Genetics, North Carolina State University, Raleigh, NC, USA; 3 Department of Entomology, Center for Pollinator Research, Center for Chemical Ecology, Huck Institutes of the Life Sciences, Pennsylvania State University, University Park, PA, USA; 4 Syngenta Biotechnology, Incorporated; Research Triangle Park, NC, USA

## Abstract

**Background:**

Regulation of worker behavior by dominant queens or workers is a hallmark of insect societies, but the underlying molecular mechanisms and their evolutionary conservation are not well understood. Honey bee and bumble bee colonies consist of a single reproductive queen and facultatively sterile workers. The queens' influences on the workers are mediated largely via inhibition of juvenile hormone titers, which affect division of labor in honey bees and worker reproduction in bumble bees. Studies in honey bees identified a transcription factor, *Krüppel-homolog 1 *(*Kr-h1*), whose expression in worker brains is significantly downregulated in the presence of a queen or queen pheromone and higher in forager bees, making this gene an ideal candidate for examining the evolutionary conservation of socially regulated pathways in Hymenoptera.

**Results:**

In contrast to honey bees, bumble bees foragers do not have higher *Kr-h1 *levels relative to nurses: in one of three colonies levels were similar in nurses and foragers, and in two colonies levels were higher in nurses. Similarly to honey bees, brain *Kr-h1 *levels were significantly downregulated in the presence versus absence of a queen. Furthermore, in small queenless groups, *Kr-h1 *levels were downregulated in subordinate workers with undeveloped ovaries relative to dominant individuals with active ovaries. Brain *Kr-h1 *levels were upregulated by juvenile hormone treatment relative to a vehicle control. Finally, phylogenetic analysis indicates that KR-H1 orthologs are presence across insect orders. Though this protein is highly conserved between honey bees and bumble bees, there are significant differences between orthologs of insects from different orders.

**Conclusions:**

Our results suggest that *Kr-h1 *is associated with juvenile hormone mediated regulation of reproduction in bumble bees. The expression of this transcription factor is inhibited by the queen and associated with endocrine mediated regulation of social organization in two species of bees. Thus, KR-H1 may transcriptionally regulate a conserved genetic module that is part of a pathway that has been co-opted to function in social behavior, and adjusts the behavior of workers to their social environmental context.

## Background

With recent advances in genomics, it is becoming increasingly feasible to elucidate the molecular mechanisms underlying complex behavioral traits in ecologically relevant contexts. One of the key challenges is to determine the level of conservation of genes and genetic pathways involved in producing or regulating similar adaptive behaviors in evolutionarily distinct lineages (reviewed in [1]). Social insects provide an excellent model system for these studies, because sociality has evolved independently multiple times [2]. One of the hallmarks of insect societies is a reproductive division of labor, in which reproduction is strongly biased toward one or a handful of individuals in the colony (queens) whereas most other colony members (workers) are engaged in very little reproduction or remain sterile. A second important element is a division of labor among the sterile workers, in which workers specialize in various activities such as brood care or foraging (reviewed in [2,3]). In most insect societies, the interactions between the queen(s) and workers are critical for regulating these divisions of labor. In simple societies, direct contact with the queen and behavioral aggression is often important for inhibiting worker reproduction, while in larger and more evolutionarily derived societies, chemical communication via pheromones is the principle means for advertising the queen's presence [2-5].

Socially regulated behavioral pathways, hereafter referred to as "social pathways", presumably link social signals from the queen and other workers with alterations in worker physiology, which in turn lead to changes in behavioral state (e.g. worker task) that are integrated into an emerging colony level function. These social pathways need to include sensory and integrative systems that ensure that the individual is tuned to the relevant social signals, as well as neuroendocrine signaling systems that integrate the molecular and physiological processes needed for producing the appropriate social behavior. One of the important challenges in sociobiology is to characterize these pathways and compare their structure and function in species showing different levels of social organization. Previous studies in behavioral genomics have focused on genes that detect a particular signal, such as a pheromone, or genes that are involved in the expression of a particular behavior, such as foraging (reviewed in [1]). While there is ample evidence that the social environment can have significant effects on behavior, physiology, and brain gene expression in a variety of species, genes involved in the intervening "social pathways" have been characterized in only a few species (reviewed in [6]). For example, the serotonergic system is modulated in crustaceans according to dominance rank, while somatostatin and gonadotropin releasing hormone levels are associated with dominance rank in male cichlid fish [7-10]. In social insects, allelic differences at the *Gp-9 *locus in fire ants are associated with differences in colony structure, but the number of linked genes in this locus and their molecular functions are not known [11].

Honey bees and bumble bees are excellent species for comparative studies of this kind, since there are both common elements and important differences in their social organization [2,3]. Honey bees are highly eusocial insects living in perennial colonies containing several tens of thousands of worker bees. The queen signals her presence by means of a complex set of pheromones that regulate the function and reproduction of the colony [12]. The division of labor among workers is related to age, and is regulated in part by queen pheromones [13]. Bumble bees such as *Bombus terrestris *are considered "primitively eusocial" because there are no overt morphological differences between the queen and the workers, and the division of labor is less structured [3]. Colonies are founded by a single queen in the spring, and reach a maximal size of only a few hundred workers. Direct contact with the queen is required to inhibit worker reproduction, though this typically does not seem to require aggressive interactions [14,15]. There is evidence which supports the hypothesis that pheromonal queen signals are involved in the regulation of worker reproduction, but the relative importance of chemical and behavioral signals has not been resolved [16-18]. Worker division of labor between brood care and foraging is based largely on body size rather than on age [2,3].

Neuroendocrine analyses suggest that there are similarities between the social regulation of reproduction in bumble bees and division of labor in honey bees, specifically concerning the role of juvenile hormone [19]. In honey bees, exposure to queen pheromone decreases circulating levels of juvenile hormone (JH), and slows the transition from nursing to foraging [13,20]. Forager bees have higher circulating levels of JH, and treatment with JH or JH analogues accelerates the transition to foraging, while JH depletion delays the onset of foraging [21,22]. In bumble bees, queen presence or body extracts also reduces JH levels. High JH levels are associated with worker ovary activation and reproduction, and high dominance rank in queenless workers [17,23-25]. JH treatment of workers causes a dose-dependent increase in oocyte length, even in the presence of an inhibitory queen, but does not affect worker task (division of labor) [25-28]. Thus, in bumble bees as in most insects, JH levels are associated with ovary activation and reproduction, while in honey bees and some ant species, juvenile hormone levels are not higher in reproductive queens or workers (for a recent review see [29]). Thus, this hormonal factor is associated with different behavioral outcomes, but in both cases JH levels are regulated by queen presence. Given that stimuli produced by the queen lead to similar changes in worker physiology but different types of behavioral outcomes, it is possible that the first portions of the "social pathway" are conserved in honey bees and bumble bees, but diverge in terms of the downstream pathways regulating behavior.

Previous studies in honey bees identified a transcription factor, *Krüppel homolog 1 *(*Kr-h1*), whose expression in the brains of workers is consistently downregulated by exposure to the queen or queen pheromone [30-32]. As expected, since queen pheromone slows the transition from nursing to foraging, expression of *Kr-h1 *is also high in foragers compared to nurse bees [31]. Thus, *Kr-h1 *expression levels may be associated with detection of queen presence, the transition from nursing to foraging, or some aspect of the foraging behavioral state, such as high levels of JH. Furthermore, as a transcription factor, *Kr-h1 *regulates expression of other genes, and thus may be a key component of signaling pathways linking social environment with behavioral and physiological state.

Here, we tested whether the association of *Kr-h1 *with a social pathway linking the processing of queen signals with JH regulated behavior is common to social bee species other than the honey bee. Using bumble bees enabled us to uncouple the molecular pathways associated with social inhibition (similar in the honey bee and the bumble bee) from those regulating behavior (distinct in the two species). For example, if *Kr-h1 *is linked to foraging, then expression should be higher in foragers than nurses in bumble bees as it is in honey bees. If *Kr-h1 *is associated with queen presence/social inhibition, then expression levels in *Bombus *should be similar in nurses and foragers, lower in queenright vs queenless workers, and lower in subordinate relative to dominant workers. Furthermore, since JH is linked with foraging behavior in honey bees but with reproductive behavior and dominance rank in bumble bees, these studies will allow us to determine if *Kr-h1 *expression is correlated with JH levels in both species, regardless of the behavioral state. Finally, we ascertained whether KR-H1 was present and conserved across insect orders. Sequence differences in transcription factors may alter the timing, location, or types of genes regulated by these proteins, and may be an important factor contributing to behavioral or phenotypic evolutionary change [33].

## Results

### The influence of queen presence on brain *Kr-h1 *levels in workers

**Figure 1:**
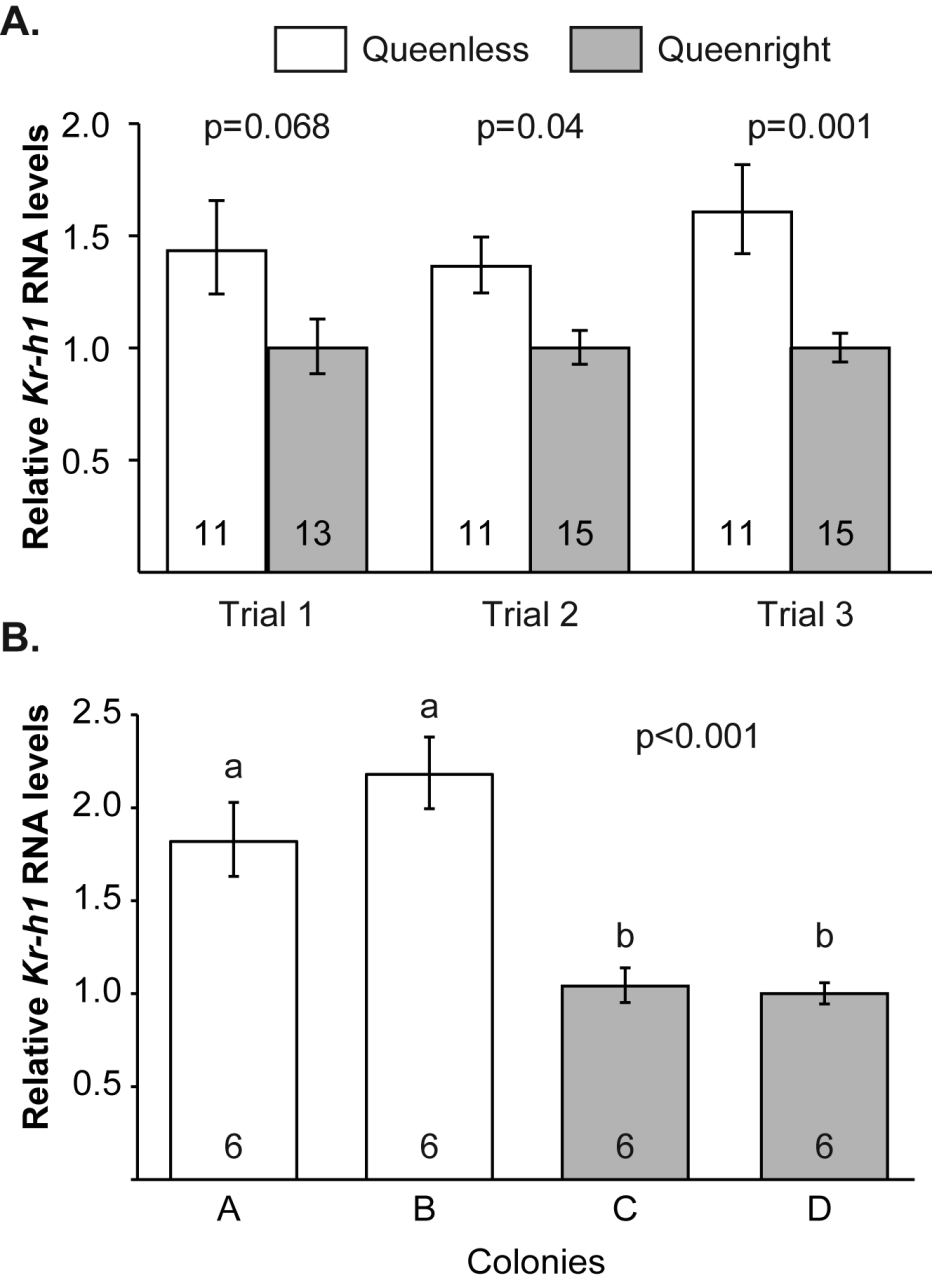
**The influence of queen presence on brain *Kr-h1 *levels in workers**. **A**. In the first experiment we compared workers from queenright colonies and workers of similar age and genotype that developed in small queenless groups. Data represent mean ± standard error of mean. The number of individual brains used in each sample is shown in the base of each bar. P-values above plots were obtained from unpaired t-test analyses. **B**. In the second experiment we compared queenless and queenright workers that developed in colonies with similar worker and brood populations, but differing in the presence or absence of an egg-laying queen. Sample size was 6 bees/colony. Groups with different letters are significantly different in a one-way ANOVA followed by a LSD post hoc test (P < 0.001 for both tests).

We performed two experiments to test the influence of the queen on brain *Kr-h1 *expression levels in workers. For the first experiment, we performed three replicates comparing queenright workers in colonies and queenless workers that developed in cages in groups of three. Brain *Kr-h1 *levels were significantly lower in queenright workers in the second and third trial (t(24) = -2.64, P = 0.014 and t(24) = -3.68, P = 0.001 respectively; Fig. 1A); a similar, but statistically not significant trend was obtained in the first trial (t(22) = -1.92; P = 0.068). There was a significant difference between queenright and queenless workers in a pooled analysis that included bees from all three trials (mixed model ANOVA, F = 21.05, df = 1, 72; P < 0.0001).

In the second experiment, queenless workers were obtained from orphan colonies, with similar results as above. The workers from the two queenless colonies had approximately twice the amount of brain *Kr-h1 *RNA compared to the workers from queenright colonies (Fig. 1B; one way ANOVA, P < 0.001; LSD post hoc test, P < 0.001). The queenless and queenright workers were of similar age (4-day-old) and size (queenless colony A, mean ± SE marginal cell size = 2.88 ± 0.12; queenless colony B, 2.94 ± 0.06; queenright colony C, 2.77 ± 0.13; queenright colony D, 2.90 ± 0.03; one-way ANOVA, F = 0.55, P = 0.65). These experiments indicate that the brain expression of *Kr-h1 *is reduced in the presence of a vital egg-laying queen.

### The influence of worker task on brain *Kr-h1 *levels

**Figure 2:**
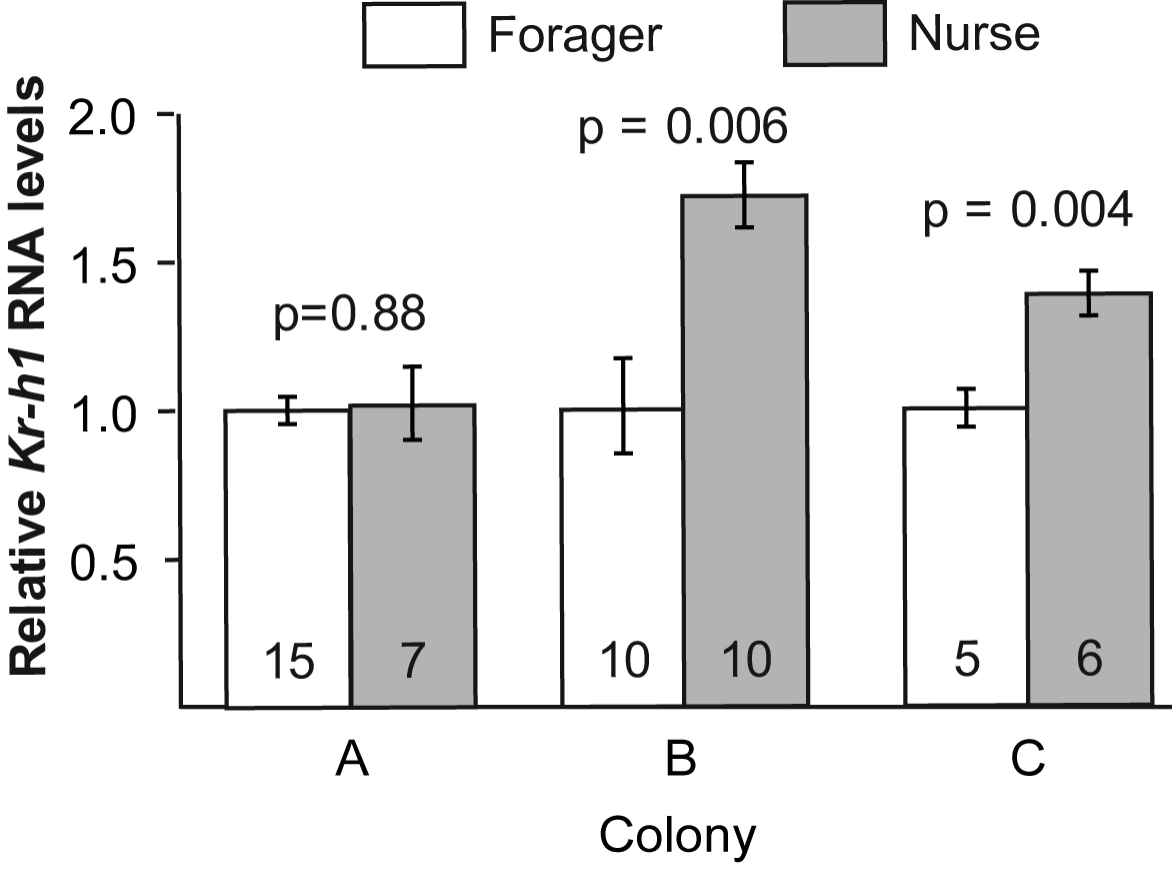
**The influence of task on brain *Kr-h1 *levels**. We collected nurses and foragers from self-supported free-flying colonies. Brain *Kr-h1 *transcript levels were similar in foragers and nurses from Colony A but higher in nurses in colonies B and C. Data represent mean ± standard error of the mean. The number of individual brains used in each sample is shown in the base of each bar. P-values above plots were obtained from unpaired t-test analyses.

We repeated this experiment with three different colonies. In colony A, *Kr-h1 *transcript levels were similar in the brains of foragers and nurses (Fig. 2A, t(20) = 0.15, P = 0.88). In colonies B and C nurse bees had significantly higher brain *Kr-h1 *levels (Fig. 2B, C; t(18) = 3.14, P = 0.006; t(9) = 3.85, P = 0.004, respectively). A two-way ANOVA with data from the three colonies pooled together produced a significant effect for task, [F = 12.1, P = 0.01], and task × colony [F = 4.08, P = 0.023]). Nurses and foragers did not differ in age (colony A, foragers, mean age = 15.2 days, range = 13-18 days of age; nurses, mean = 14, range 13-15; t-test t(20) = 1.75 P = 0.094; colony B, foragers, mean = 17.1, range = 9-28; nurses, mean = 24, range 14-35; t-test, t(18) = 1.896, P = 0.075; Colony C, foragers, mean = 5, range 3-7; nurses, mean 4.5 range 3-8; Mann-Whitney U test, two-tail, P = 0.5). There were no significant differences in body size but there was a trend toward larger size for foragers in colony C (Colony A, wing length [mean ± SE], foragers = 11.84 ± 0.37 mm, nurses = 11.43 ± 0.48 t-test t(20) = 0.66, P = 0.52; Colony C, marginal cell length, foragers = 2.92 ± 0.09 mm, nurses = 2.71 ± 0.07; Mann Whitney U test, two-tail, P = 0.1). The ovaries were in basal state for all bees in the two groups except for one nurse in colony A with a largest oocyte = 2.5 mm. The basal state of the ovaries was probably because these bees were queenright [24,28,34]. These results indicate that by contrast to honey bees, foraging behavior is not associated with elevated brain *Kr-h1 *RNA levels. Indeed, in 2 out of 3 colonies, nurses have higher levels of brain *Kr-h1 *RNA than foragers.

### The influence of dominance rank on *Kr-h1 *levels in groups of queenless workers

**Figure 3:**
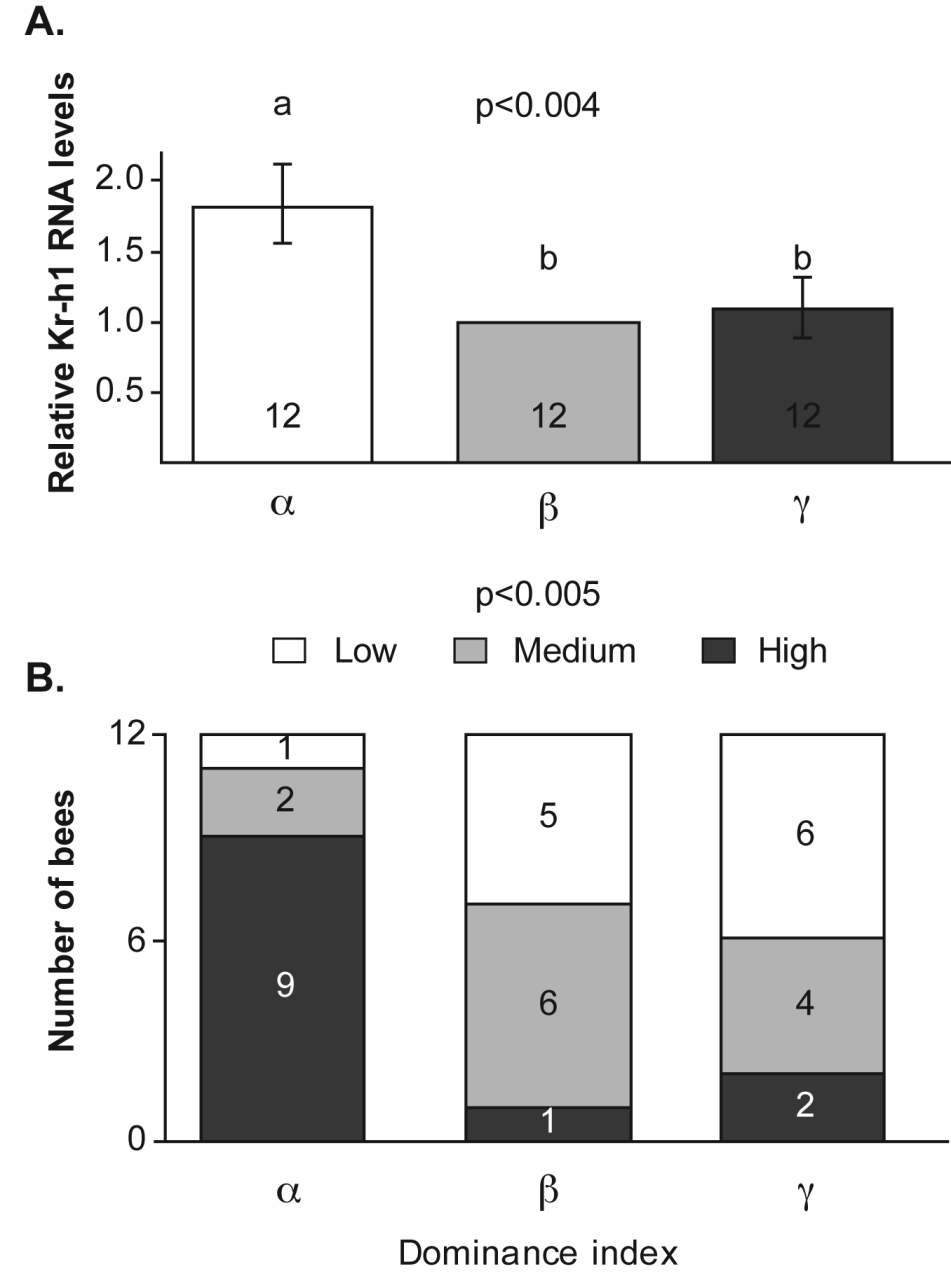
**The influence of dominance rank on brain *Kr-h1 *levels in groups of three queenless workers**. **A**. Average brain *Kr-h1 *RNA levels for bees with different dominance rank. Data represent mean ± standard error and the sample size is indicated within bars. The P-value was obtained from a one way ANOVA. Groups with different letters are significantly different in a LSD post hoc statistical test (P < 0.05). **B**. The proportion of bees in each dominance rank with the highest, medium, and lowest brain *Kr-h1 *RNA levels. The numbers inside the bars show the number of bees fitting each category. The P-value above plot shows the result of a Chi square goodness of fit test comparing the observed values to an expected probability of 0.33 if dominance and *Kr-h1 *levels are independent.

Groups of three queenless workers were reared in cages and allowed to establish dominance hierarchies. We observed frequent agonistic interactions on days 3 and 7, enabling a clear sorting of the dominance order for 12 out of 13 queenless groups. Only these 12 groups were used for the analyses summarized below. The α bee was the most aggressive, had an average dominance index of 0.93, and could be clearly distinguished from the two subordinate bees in all 12 groups. The β and γ bees, with an average dominance index of 0.37, and 0.06, respectively, were in some groups harder to differentiate either because they performed only a few social interactions, or both subordinate bees retreated in almost all their encounters. Brain *Kr-h1 *RNA levels for bees collected at seven days of age were correlated with the dominance rank. An ANOVA revealed significantly higher brain *Kr-h1 *levels for the most dominant individual (α), compared with the β and γ subordinate groupmates (Fig. 3A, one way ANOVA, F = 6.72, df = 2, P = 0.004, LSD post hoc test, P < 0.05). In 9 of 12 groups, the most dominant individual also had the highest *Kr-h1 *brain RNA levels, which is significantly different from the expected 0.33 probability (4 groups) of independence for the two traits (Fig. 3B, χ^2 ^= 15 df = 4, P = 0.005). The bee with the highest *Kr-h1 *levels always had developed ovaries, even in groups in which she did not hold the highest dominance rank. This experiment suggests that brain *Kr-h1 *levels are relatively low in bees for which ovarian development is inhibited by the presence of a dominant groupmate.

### The influence of juvenile hormone on ovarian development and brain *Kr-h1 *levels in queenright workers

Two trials were completed for this experiment. Measurements for the length of the front wing marginal cell indicate that there were no differences in body size between the three experimental groups (Kruskal Wallis Test; first trial: χ^2 ^= 0.38, df = 2, P = 0.82; second trial: χ^2 ^= 4.512, df = 2, P = 0.1, Table 1). Ovaries at seven days of age were at a significantly more advance stage of development for the JH treated bees in the second trial (Table 1; Kruskal Wallis Test, χ^2 ^= 13.45, df = 2; P = 0.0012), and a similar trend was also seen in the first trial in which sample size was smaller, although the results were not statistically significant (χ^2 ^= 3.99, df = 2, P = 0.14). This analysis of ovarian state is consistent with previous JH-treatment studies [25,26].

**Table 1:** The influence of topical juvenile hormone (JH-III) treatment on ovarian development in queenright workers.

		JH-III	DMF	Control	Kruskal Wallis test
**Trial 1**	Length of marginal cell (mm)	2.72 ± 0.15 (5)	2.68 ± 0.16 (6)	2.68 ± 0.11(4)	**0.83**
	
	Length of terminal oocyte (mm)	1.90 ± 0.41(5)	1.27 ± 0.23 (5)	0.93 ± 0.09 (4)	**0.14**

**Trial 2**	Length of marginal cell (mm)	2.74 ± 0.06 (8)	2.74 ± 0.06 (8)	2.89 ± 0.08 (7)	**0.1**
	
	Length of terminal oocyte (mm)	2.41 ± 0.27 (8)	1.08 ± 0.15 (8)	1.20 ± 0.18 (7)	**0.001**

One-day-old workers were topically treated with 70 μg juvenile hormone (JH-III) dissolved in 5 μl DMF, 5 μl DMF vehicle (DMF), or were similarly handled but not treated (Control). We measured the length of the marginal cells and of terminal oocytes of these bees on day 7. The table shows mean values ± standard error of the mean, sample size in parentheses (n). The P-values of the Kruskal Wallis tests are shown. DMF = Dimethylformamide.

JH treated bees in the first trial had significantly higher brain *Kr-h1 *RNA levels compared to bees from the DMF group, but not from the control group (Table 2, one way ANOVA, F = 4.64, df = 2, P = 0.018, LSD post hoc test, P < 0.05). In the second trial, *Kr-h1 *levels were significantly higher in the JH treatment group compared to the two other groups (Table 2; one way ANOVA, F = 9.01, df = 2, P = 0.001; LSD post hoc test, P < 0.05). The effect of treatment (P = 0.003) and colony × treatment (P = 0.032), but not of the colony (P = 0.14) was significant in a two-way ANOVA with bees from the two trials pooled together. Complementary LSD post hoc tests revealed a highly significant difference between the JH and DMF treatments (P = 0.0004), and a very strong trend for JH vs. control (P = 0.057). The consistent influence of JH treatment relative to the vehicle (DMF) control suggests that *Kr-h1 *expression is up-regulated by JH in *Bombus terrestris*.

**Table 2:** The influence of topical juvenile hormone treatment on brain *Kr-h1 *levels.

	JH-III	DMF	Control	ANOVA
**Trial 1**	1.65 A (0.21, 0.18, 10)	1.00 B (0.12, 0.11, 10)	1.63 A (0.28, 0.24, 10)	**P = 0.018**

**Trial 2**	1.58 A (0.08, 0.08, 9)	1.12 B (0.12, 0.11, 8)	1.00 B (0.09, 0.09, 9)	**P = 0.001**

Bees were treated as described in Table 1. We measured brain Kr-h1 RNA levels for these bees on day 3. Data represents mean, in parentheses the positive standard error of mean, negative standard error of mean, and sample size, respectively. Means with different capital letters are significantly different in a LSD post hoc statistical test (P < 0.05).

### Analysis of KR-H1 orthologs across insect orders

**Figure 4:**
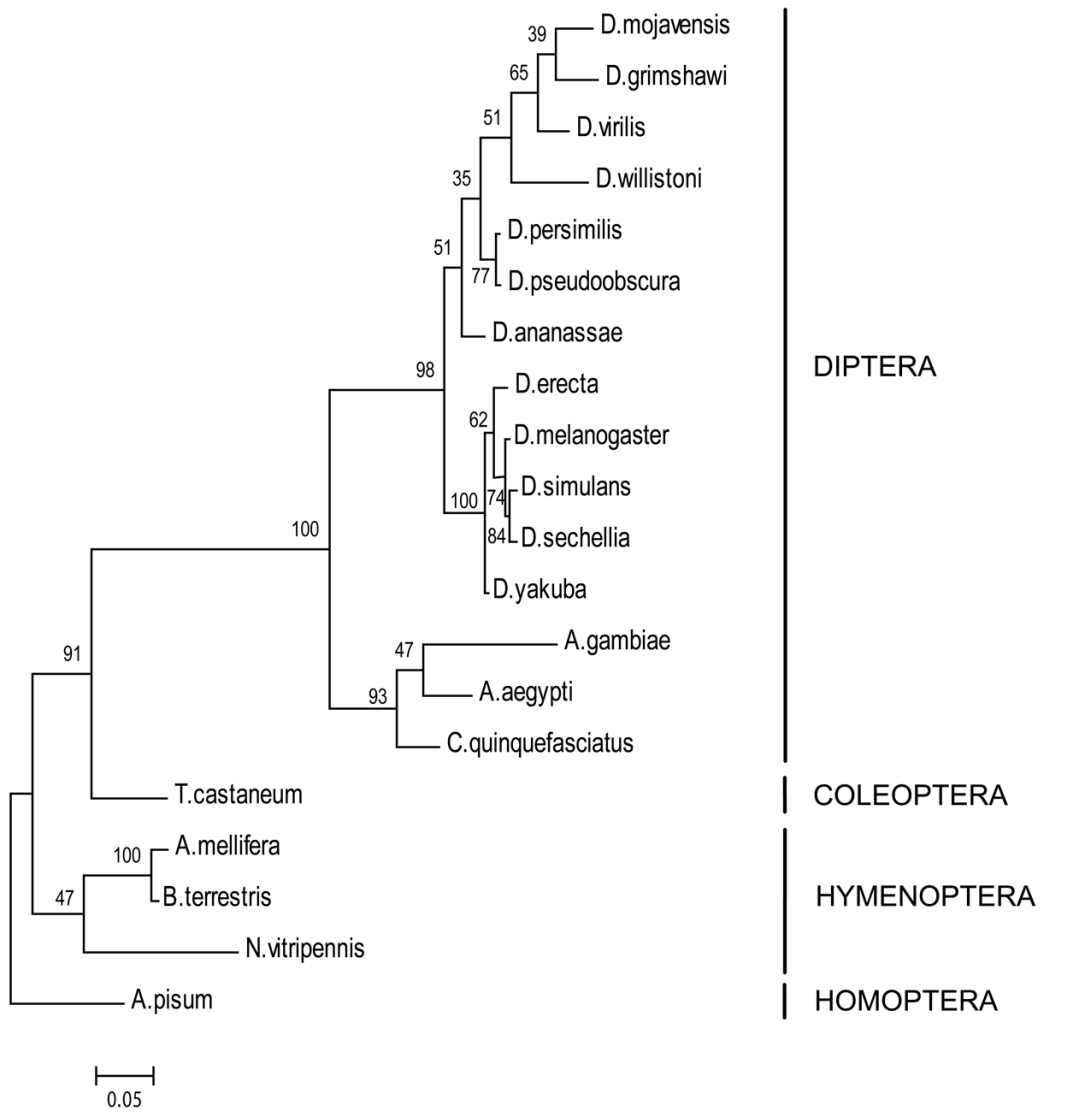
**Phylogenetic tree of *Kr-h1 *orthologues**. A maximum likelihood phylogenetic tree examining the relationship between the BtKR-H1 ortholog and orthologs from 19 other insect taxa, using the conserved zinc finger region (see Additional file 1,2 Fig. S1). Maximum likelihood bootstrap values in percent (1000 replicates) are shown at each node. Branch lengths are from maximum likelihood. Insect orders are indicated to the right. The hemimetabolous insect *A. pisum *is set as the outgroup to the holometablous insects. *B. terrestris *and *A. mellifera *are shown within the Hymenoptera. The GenBank accession numbers and full names of all species can be found in the methods.

KR-H1 orthologs were identified in the 20 insect species with available genomic information. Alignments of the full protein sequences of the orthologs revealed that the zinc-finger DNA binding domains were conserved as were two regions in the C-terminal domain, but otherwise there was a great degree of sequence divergence (see Additional files 1, 2 Fig. S1 for alignments of orthologs). KR-H1 orthologs are characterized by eight classic C_2_H_2 _zinc fingers described as CX_2_CX_12_HX_3_H. These types of proteins have a complex binding capacity: similar multiple-adjacent C_2_H_2 _proteins such as TFIIIA, WT1 and Roaz bind only 24-75% of their zinc fingers to DNA, while the remaining zinc fingers may bind to proteins and RNA, including dsRNA and DNA-RNA heterocomplexes (see [35] for review). A phylogenetic maximum likelihood analysis using the zinc finger regions for the predicted KR-H1 proteins is not in conflict with the phylogenetic relationship of the represented insect orders (Fig. 4) albeit with weak bootstrap support [36,37]. The KR-H1 tree should not be regarded as an approximation of insect species phylogeny: it is intended to represent the relative relationship of the KR-H1 orthologs of *A. mellifera *and *B. terrestris *to each other and to species from other orders of insects.

There is a high degree of conservation between the predicted *B. terrestris *KR-H1 protein and the *A. mellifera *protein, with 97% identity across the whole protein without gaps removed. Interestingly, the sequence of the ortholog from *Nasonia vitripennis*, a solitary wasp and the only other hymenopteran ortholog currently available, is highly divergent from the two bee species in the C-terminal region. Data from other species will be necessary to determine if this sequence divergence is related to differences in evolutionary lineage or associated with differences in life history or behavior.

While the zinc finger regions are conserved among the insect KR-H1 orthologs, there are important variations in the arrangement of these zinc fingers between the lineages, particularly between dipteran and non-dipteran species, which may have consequences on KR-H1 function. Most importantly, the coding region between zinc finger 1 (Z1) and Z2 contains an insertion-deletion (indel) that varies with taxon. In the dipeteran lineage, the indel ranges from 34-65 aa, and thus is much longer than in other taxa, where the length varies from 9-14 aa. The other zinc fingers are universally separated by 7 residues. In addition to the zinc finger region, there is a conserved region at the C-terminus of KR-H1 orthologs, characterized by LP(L/P)RKR which is separated by a variable indel from RX_2_SVIX_2_A at the extreme C-terminus. In the dipteran lineages, the indel ranges from 28 to 60 aa, and thus is much longer than in other taxa where the length varies from 14 to 19 aa.

## Discussion

Regulation of worker behavior and physiology by dominant queens and workers is a hallmark of insect society. In honey bees and bumble bees, this regulation is largely mediated by inhibition of JH levels in workers, which results in inhibition of behavioral maturation in honey bees and reproductive development in bumble bees [13,19,23]. These observations support a model in which social stimuli inhibit similar JH-mediated social pathways in both species, but these pathways ultimately regulate different behavioral programs. Together, the studies in honey bees and bumble bees suggest that *Kr-h1 *is a part of a conserved genetic module which was co-opted to adjust the social behavior of workers to their social environmental context.

In the bumble bee, brain *Kr-h1 *levels are associated with dominance and reproductive status. *Kr-h1 *levels are high in the brains of dominant queenless bees, which are characterized by rapid oogenesis and high JH titers. Brain *Kr-h1 *levels are low in queenright workers and subordinate queenless workers in which oogenesis is inhibited by the presence of a dominant queen or worker [23-25,34,38]. In contrast to honey bees, high *Kr-h1 *levels were not associated with foraging behavior. In fact, in two of the colonies *Kr-h1 *levels were higher in nurses than in foragers, which is consistent with the general association between high *Kr-h1 *levels and ovarian activity: oogenesis is typically more rapid in nurses than in foragers of similar age and genotype [34,28,39]. However, in both of these groups ovarian development is slower than in queenless workers (see above) which may account for the overall low ovarian activation measured for 7-day-old bees from colonies B and C. The association between *Kr-h1 *expression, JH, and ovarian activity is further exemplified by the evidence that JH treatment caused an increase in both *Kr-h1 *expression and ovarian development.

In honey bees, the regulation of *Kr-h1 *is different. Brain *Kr-h1 *levels were not significantly different between workers with activated or inactivated ovaries, and *Kr-h1 *levels were not regulated by mating or reproductive status in queens [40,41]. While brain *Kr-h1 *levels are always higher in foragers relative to bees involved in in-hive activities, *Kr-h1 *levels were not significantly different between foragers and foragers that reverted to performing nursing behavior [31,42]. Similarly, there is increased neural branching in the mushroom bodies which develops when the bee becomes a forager but does not change when she reverts to brood care [43]. JH treatment affected this processes in some studies, but neuronal branching did occur in bees with no circulating JH, suggesting that JH may affect the pace of mushroom body development, but not its overall occurrence [44]. In this regard, it is interesting to note that there is some evidence that *Kr-h1 *may play a role in modulating neuromorphology in *Drosophila*, and expression levels of *Kr-h1 *are specifically regulated in the mushroom bodies of honey bee workers [32,45]. These recent studies with honey bees lend credence to the premise that *Kr-h1 *is more strongly associated with the detection and processing of social stimuli than specifically with foraging behavior or reproduction.

The correlation between JH titers and brain *Kr-h1 *levels under diverse social conditions in both honey bees and bumble bees suggest that *Kr-h1 *and JH are involved in the same pathway (or network) that is influenced by signals from the queen and other workers. In the bumble bee *B. terrestris*, JH treatment caused a significant increase in brain *Kr-h1 *transcript levels relative to a vehicle treatment, suggesting that JH acts upstream of *Kr-h1*. This model is consistent with findings in *D. melanogaster *and *Tribolium casteneum*, in which *Kr-h1 *expression is increased by treatment with JH analogs during prepupal and pupal stages [46-48]. In honey bees, it is more difficult to precisely determine the relative position of JH and *Kr-h1 *in the signaling pathway, and the relationship between these two factors may be more complex. Treatment with the juvenile hormone analog methoprene increased brain *Kr-h1 *levels in queenright bees [31]. By contrast, in queenless groups of bees, neither methoprene nor JH III treatment raised *Kr-h1 *levels above those found in control bees (Fussnecker and Grozinger, unpublished observations), and *Kr-h1 *levels are not always high when JH levels are high [42]. One interpretation for this apparent discrepancy between the treatment effect in queenright and queenless conditions is that JH does influence *Kr-h1 *expression, but under queenless conditions transcript levels are already high and cannot be increased further by treatment with JH or its analogs. Alternatively, *Kr-h1 *expression may be regulated by additional factors that mask the influence of JH. The influence of JH treatment on queenless workers has not yet been tested for bumble bees, and thus the relationship between *Kr-h1 *levels and JH titers in bumble bees might also be affected by social conditions.

Transcription factors like *Kr-h1 *play an important role in the evolution of physiological, morphological, and behavioral traits [33]. Changes in the amino acid sequence - including the addition or deletion of small protein-binding motifs or alterations in the arrangement of DNA-binding domains - could modulate the function of transcription factors, and the modular architecture of these proteins suggest these changes can occur without large pleiotropic consequences. This sequence variation could alter the types of promoter regions that transcription factors bind to, or modify protein-protein interactions, leading to differences in the signaling pathways that regulate transcription factors. *Kr-h1 *plays an important role during development and metamorphosis, particularly in development of the central and peripheral nervous system in *D. melanogaster *and *Tribolium castanium *[45-49]. However, its function in Hymenoptera at some level may be quite divergent, particularly given the significant sequence differences between dipteran and non-dipteran KR-H1 orthologs. Furthermore, the molecular function of *Kr-h1 *in adult insects remains to be determined. It is interesting to note that while the *B. terrestris *and *A. mellifera *orthologs are nearly identical there are large differences in the C-terminal region of the predicted *Kr-h1 *ortholog from *N. vitripennis*, a solitary parasitic wasp. Further comparative genomic studies and functional assays will be necessary to determine if *Kr-h1's *function truly diverges between social and nonsocial Hymenoptera, and between different insect orders, or if the sequence differences are simply due to the accumulation of mutations during evolution in different lineages.

## Conclusions

Our results suggest that *Kr-h1 *is associated with JH mediated regulation of reproduction in bumble bees. In contrast, in honey bees *Kr-h1 *is associated with worker division of labor. In both bee species however, the expression of *Kr-h1 *in the brain is inhibited by the queen and associated with juvenile hormone mediated regulation of social organization. The comparative studies with honey bees and bumble bees suggest that *Kr-h1 *is part of a conserved pathway linking the perception of social signals from the queen and other workers to JH regulated changes in social behavior. Since the KR-H1 orthologs in honey bees and bumble bees are highly conserved, they may transcriptionally regulate similar genetic modules. However, we hypothesize that downstream of *Kr-h1*, the pathway may diverge to regulate functions associated with worker task in honey bees and reproduction in bumble bees. Additional studies on the function of *Kr-h1 *and the genes it regulates may allow a better understanding of the evolution of queen control over worker behavior in social insects.

There is increasing evidence that the genetic pathways underlying developmental, physiological, or behavioral traits are conserved across species, and modifications in the coordination or expression of these pathways can have profound effects and produce distinct phenotypes (reviewed in [50,51]). Comparisons of honey bees and bumble bees allow us to examine the genetic conservation of socially responsive behavioral pathways, and to elucidate the interplay of specific genes and physiological pathways, such as JH. JH is a principle insect endocrine signal that appears to have a somewhat variable function in social insects. It retains its "classical" gonadotrophic function in some species such as the bumble bee *B. terrestris*, while in other species such as the honey bee *A. mellifera *it is instead implicated in the regulation of age-related division of labor [29,52-54]. Given of the association of social organization, JH levels, and *Kr-h1 *expression, additional studies on the function of *Kr-h1 *and the genes it regulates in bees and other insect species will allow us to further characterize this pivotal "social pathway", and ultimately shed light on the evolution of complex insect societies and their enigmatic endocrine regulation.

## Methods

### General Bumble Bee Rearing

We obtained colonies of *Bombus terrestris *from Polyam Pollination Services, Yad-Mordechai, Israel. Each colony contained a queen, 5-10 workers (about 3 - 7 days after the emergence of the first worker), and brood at different stages of development. Colonies were housed in wooden nesting boxes (30 × 23 × 20 cm) with Plexiglas covers in an environmental chamber (28 ± 1°C; ~50% RH) in constant darkness at the bee research facility at the Edmond J. Safra campus of the Hebrew University of Jerusalem, Givat Ram, Jerusalem. Commercial sugar syrup and fresh pollen (mixed with sugar syrup) were provided *ad libitum*. All observations and treatments were made under dim red light that the bees cannot see. As an index for the degree of ovarian activity, we measured the length of the largest terminal oocyte [24,26,34]. As an index for body size, we measured the length of the front wing margin cell, which is highly correlated with wing length and other indices for body size (e.g., [55]).

### Identification of *B. terrestris Kr-h1*

Total RNA was extracted from the heads of individual bumble bees using a PicoPure kit (Molecular Devices, Sunnyvale, CA). cDNA was synthesized using 100 ng of total RNA with ArrayScript Reverse Transcriptase (Ambion, Austin, TX). PCR primers designed to *A. mellifera Kr-h1 *(see Additional file 3 Table S1) were used in a standard PCR reaction mix which also included 5% DMSO in a touchdown PCR reaction. PCR products were separated on a 1% agarose gel. Each primer pair produced a single dominant band. Gel bands were cut for DNA purification (QIAquick gel extraction kit, Qiagen, Valencia, CA). DNA quantity and quality was assayed with a ND-1000 Spectrophotometer (NanoDrop Technologies, Wilmington DE). Sequencing was performed with the PCR primers at the NCSU Genome Research Laboratory using the BigDye Terminator sequencing protocol (Applied Biosystems, Foster City, CA). This procedure was repeated on a second *B. terrestris *sample, as well as an *A. mellifera *sample from the same apiary at HUJI, to ensure that there was no cross-species contamination and the *B. terrestris *and *A. mellifera *sequences were distinct. The *B. terrestris Kr-h1 *sequence is available in GenBank (GQ903677).

### Quantitative real-time PCR (qRT-PCR) analysis of brain gene expression

Bees were collected by flash freezing in liquid nitrogen and immediately transferred to dry ice. Heads were separated and stored at -80°C until RNA analysis. Heads were partially lyophilized to facilitate dissection of brains. The total RNA from the dissected tissue was extracted using an RNAeasy kit (Qiagen, for analyses carried out in NCSU) or Invisorb Spin Tissue RNA Mini Kit (Invitek GmbH, Berlin, Germany, for analyses carried out in HUJI), and quantified via a ND-1000 Spectrophotometer (NanoDrop Technologies). cDNA was synthesized from 250 ng of RNA. To estimate the level of gene expression we used an ABI 7900 (NCSU) or an ABI7000 (HUJI) sequence detector and a SYBR green detection protocol (Applied Biosystems). *Elongation Factor 1 a *(*EF1a*) was used as a control housekeeping gene (for more details see [32,56]). Three technical replicates were performed for each sample/primer set. Quantification was based on the number of PCR cycles (Ct) required to cross a threshold of fluorescence intensity, using the 2^-^^Δ^^Ct ^technique (ABI User Bulletin 2). For graphical representation, data were normalized to the sample with the lowest relative *Kr-h1 *levels (which therefore received the value 1), and fold differences in relative expression levels are depicted as mean ± se. For the statistical analyses we used the ΔCt values, which are normally distributed. The sequences of the *Kr-h1 *and *EF1a *primers are in Additional file 4, Table S2. We used 6-15 biological replicates for each sample group; the sample size for each experiment is presented in the Results section.

### The influence of queen presence on brain *Kr-h1 *levels in workers

We carried out two experiments to test the influence of the queen on *Kr-h1 *levels in workers. In the first experiment, we collected 1-day-old callow workers from mixed colony backgrounds (this is done because a single colony does not produce a sufficient number of newly emerged bees in a single day), paint-marked them on their thoraces, and assigned them randomly to one of the following social environments: 1) a queenright colony, or 2) orphan groups of three workers in wooden cages (10 × 11 × 5 cm) with a Plexiglas top (3 workers/cage). We placed the colonies and small groups in an environmental chamber (28 ± 1°C) and provisioned them with commercial sugar syrup (Polyam, Yad Mordechai, Israel) and ground pollen. Maintaining workers in small groups in cages mimics the social environment of a queenless colony but allows a higher proportion of workers to acquire the top (α) dominance rank [24,26,34,57]. Using multiple small queenless groups therefore results in the production of a higher proportion of uninhibited bees, because dominant workers typically inhibit reproductive physiology and behavior in subordinate groupmates [25,26,38,57]. Workers from the queenless groups and from the queenright colonies were collected after 4 days into liquid nitrogen. Previous studies indicate that at four days of age there are significant physiological and behavioral differences between queenless and queenright workers [23-26,34,58,59]. Three trials were completed, for a total of 43 queenright and 33 queenless individuals. Brain *Kr-h1 *levels were quantified at NCSU using qRT-PCR, as described above. Data from individual trials were analyzed using a two-tailed t-test; data from all three trials were combined and analyzed in a mixed model ANOVA (proc mixed in SAS 9.2, SAS Institute Inc, Cary, NC) with treatment and trial as variables.

In the second experiment we compared workers that were reared in queenless and queenright colonies. We obtained 4 colonies, each with about 20 workers and a similar amount of developing brood. After one day of acclimation to the lab, we removed the queen from two randomly selected colonies, and within two hours introduced 12 callow bees (from a mixed origin pool of bees that emerged during the previous 24 hours) into each colony. In order to keep colony size and density intact, we removed a similar number of workers from each host colony [60]. The bees were collected and analyzed as described above. Brain *Kr-h1 *levels were quantified at HUJI using qRT-PCR. For statistical analysis we used a one-way ANOVA with colony as a variable (SPSS 15.0).

### The influence of task on brain *Kr-h1 *levels

We placed incipient bumble bee colonies in an environmental chamber (28 ± 1°C). During the first one to three weeks the colonies were fed *ad libitum *with commercial syrup and fresh pollen (collected by honey bees). The nest box was connected to the outside by a clear plastic tube (~1 m length, 2 cm diameter) and food provisioning was gradually stopped. We performed three repetitions, each with a different colony (termed A, B, and C). We connected the colonies when they contained ~50 workers (Colony A, 18 days from the emergence of the first worker), ~70 workers (Colony B, 19 days after the emergence of the first worker), or 20 workers (Colony C, about two weeks after the emergence of the first worker). We performed detailed (1-2 hrs during the morning, and 1-2 hrs during the evening, at a similar time of day) daily observations, starting three to seven days after connecting the colony to the outside. We marked each bee with a number tag and observed the colonies for at least three consecutive days before collection. Foragers were defined as bees observed returning to the hive with conspicuous pollen loads on their hind legs (returning from a foraging trip) every day during the observation period while doing no, or very little, brood care; nurses were bees observed caring for the brood, and doing no, or very little, foraging during this period (for more details, see [55]). We collected samples for RNA analysis (nurses N = 7, 25, 19; forager N = 15, 14, and 10 from colonies A, B, and C, respectively) on the last observation day (approximately 32, 33, and 26 days after the emergence of the first worker for colonies A, B, and C, respectively). During this time the colonies were self supporting. Colonies were monitored for the remainder of their life cycle, and the presence of open egg cells was used to estimate the initiation of the "competition phase" (which is characterized by competition over reproduction between the queen and workers; [38,61]). Based on this, Colony B was likely in the competition phase at the point of collection. Data were analyzed using two-tailed t-tests, and two-way ANOVA for data pooled from the three trials. In the first repetition (Colony A), we collected the samples in Israel and shipped them on dry ice to NCSU for gene expression analysis. In the second and third repetitions (Colonies B and C) we measured the RNA levels in Israel. We measured ovarian status and body size only for bees from Colony A and C.

### The influence of dominance rank on brain *Kr-h1 *levels in groups of queenless workers

One-day old workers from mixed colony backgrounds were paint-marked and placed in groups of three in small wooden cages in a constantly dark environmental chamber (28 ± 1°C, ~50%RH). Bees in each group were of similar body size and age. For each queenless group, at least two sets of observations were carried out (20 min each): the first on day three, and the second on day seven, just before collecting the bees into liquid nitrogen. Dominance index was calculated following the method described in [24]. Briefly, for each encounter, we recorded which bee advanced and which retreated. The dominance index was defined as: 1 - retreats/total encounters. In addition we recorded all agonistic behaviors including threatening behavior and overt aggression [24,62]. We classified the bees according to their dominance rank; the most dominant bee was dubbed "α", the median "β", and the lowest in the rank "γ". Brain RNA levels were analyzed only for groups in which the degree of aggressiveness matched the calculated dominance rank (the α bee is the most agonistically active) for a total of 12 groups (out of 13 groups tested). Brain *Kr-h1 *levels were quantified in Israel using qRT-PCR, as described above. Data were analyzed using a one-way ANOVA with dominance rank as the variable, and with a Chi-square goodness of fit test with the expected probability for dominant individual to have the highest brain *Kr-h1 *levels = 0.33.

### The influence of juvenile hormone on *Kr-h1 *levels in queenright workers

One-day-old workers from a mixed colony background were paint-marked and assigned randomly to one of the following treatments (N = 15-17 bees/treatment): 1) JH-treatment, we dissolved 70 μg JH-III (Sigma, cat J2000, purity ≥ 65%, lot# = 087K26321; St. Louis, MO) in 5 μl DMF (J.T Backer cat: 7032 - 1L lot: 0509710007 Dimethylformamide) and applied the solution to the dorsal part of the thorax following the method of [63]. We used JHIII because it is the only JH analogue found in *B. terrestris *[23,24]. 2) DMF treatment, same as in (1), but we treated the bees with 5 μl DMF; 3) untreated control, we handled the bees as in the two other groups, but did not treat them with any solution. Bees from all three treatments were introduced into a young queenright colony (containing a viable queen and before the competition phase); all the introduced workers were accepted to the host colonies with no evidence for aggression. After 3 days we collected eight to ten bees/treatment into liquid nitrogen for subsequent RNA analysis. At 7 days of age, we collected five to eight additional bees per group for the analysis of ovarian state and body size. We used a mixed model ANOVA with treatment as the variable for the analyses of gene expression data and body size, and the Kruskal Wallis nonparametric test for the analysis of ovarian state (all proc mixed in SPSS 15.0).

### Alignments of *Kr-h1 *orthologs

Sequences of 20 KR-H1 insect orthologs were obtained (Additional file 1,2 Fig. S1, GenBank accession numbers for all orthologs can be found below). For the *Drosophila *species, the isoforms orthologous to *Drosophila melanogaster *KR-H1β were used. The complete protein sequences were aligned using ClustalW packaging in BioEdit 7.0, using the full multiple alignment feature (Additional file 1,2 Fig. S1)[64]. Alignment parameters were set to BLOSUM protein weight matrix with a gap opening penalty set to 10.0 and gap extension penalty set at 0.05. Sequences were not aligned by eye given the high degree of divergence outside of conserved regions.

The relatively conserved zinc finger region, which spans 277 amino acids sites and is indicated by the red bar in Additional file 1,2 Fig. S1, was used for the phylogenetic analysis. The maximum likelihood tree was constructed using phyML 3.0, under a fixed model of amino acid substitutions, allowing for substitution rate variation among site with a gamma distribution [65-67]. The tree was drawn in MEGA4 [68]. *Acyrthosiphon pisum *(Homoptera) was used as the outgroup to the holometabolous insects based on well-established species phylogenetic data [36,37,69-71]. Branch support values are indicated at each node are from an analysis of 1000 bootstrap replicates. The WAG model of amino acid evolution was used [72]. The final tree was uploaded into treeBASE (Accession number SN4664).

Accession numbers are as follows: pea aphid, *Acyrthosiphon pisum *(XM001946159); *Aedes aegypti *mosquito (EAT46451); *Anopheles gambiae *mosquito (EAA13888); honey bee, *Apis melllifera *(AAR08420); *Bombus terrestris *(GQ903677); *Culex quinquefasciatus *mosquito (EDS38735); *Drosophila ananassae *(EDV31532); *Drosophila erecta *(EDV59037); *Drosophila grimshawi *(EDW02871); *Drosophila melanogaster *(CAA06543); *Drosophila mojavensis *(EDW11744); *Drosophila persimilis *(EDW28206); *Drosophila pseudoobscura *(EAL34248); *Drosophila sechellia *(EDW54474); *Drosophila simulans *(EDX03916); *Drosophila virilis *(EDW63622); *Drosophila willistoni *(EDW76311); *Drosophila yakuba *(EDW87936); *Nasonia vitripennis *wasp (XP001600294); and the red flour beetle, *Tribolium castaneum *(AB360764).

## Authors' contributions

HS carried out most of the molecular analyses, and participated in designing the experiments and in the preparation of the manuscript. HMP carried out the sequence and phylogenetic analyses and data summary. YF contributed to the sequencing of *B. terrestris Kr-h1*. MC and YF contributed to the molecular analyses of *Kr-h1 *levels. CMG and GB designed the study and wrote the paper. All authors read and approved the final manuscript.

## Supplementary Material

Additional file 1 **Alignments of the insect KR-H1 orthologs**. Figure legend for the additional file 2 fig. S1. Click here for file

Additional file 2 **Alignments of the insect KR-H1 orthologs**. Alignment of KR-H1 orthologs from 20 insect species Click here for file

Additional file 3 **Primers for amplifying *Bombus *Kr-h1**. Sequences of the primers used for cloning of the *Bombus terrestris Kr-h1 ortholog*. Click here for file

Additional file 4 **Primer sequences for quantitative real-time PCR**. Sequences of the *B. terrestris*, Kr-h1 and EF1a primers used for qRT-PCR. Click here for file
