# Supplementary material for: The transcription factor Krüppel homolog 1 is linked to hormone mediated social organization in bees
Source: BMC Evol Biol. 2010 Apr 30;10:120. doi: 10.1186/1471-2148-10-120 (PMC2876159; doi:10.1186/1471-2148-10-120)
Supplement: Additional file 1 — Alignments of the insect KR-H1 orthologs. Figure legend for the additional file 2 fig. S1. [file 1471-2148-10-120-S1.DOC]

**Figure S1. Alignment of insect KR-H1 orthologs.** The sequences of the 20 KR-H1 orthologs were aligned using ClustalW and the figure was constructed with BioEdit 7.0.9.0. Average amino acid identity is 0.2881. Note the conserved 8 zinc finger region toward the N-terminus with variable insertion or deletion (indel) in dipterans (between zinc finger 1 (Z1) and 2 (Z2)) and the conserved LP(L/P)RKR motif (A) separated by a variable indel from RX_2_SVIX_2_A (B) at the extreme C-terminus. The red bar indicates the region used to construct the maximum likelihood tree in Figure 5. Accession numbers for all the sequences can be found in the materials and methods.
