# Supplementary material for: The transcription factor Krüppel homolog 1 is linked to hormone mediated social organization in bees
Source: BMC Evol Biol. 2010 Apr 30;10:120. doi: 10.1186/1471-2148-10-120 (PMC2876159; doi:10.1186/1471-2148-10-120)
Supplement: Additional file 3 — Primers for amplifying Bombus Kr-h1. Sequences of the primers used for cloning of the Bombus terrestris Kr-h1 ortholog. [file 1471-2148-10-120-S3.DOC]

**Table S1. Primers for amplifying *Bombus Kr-h1*.**

**
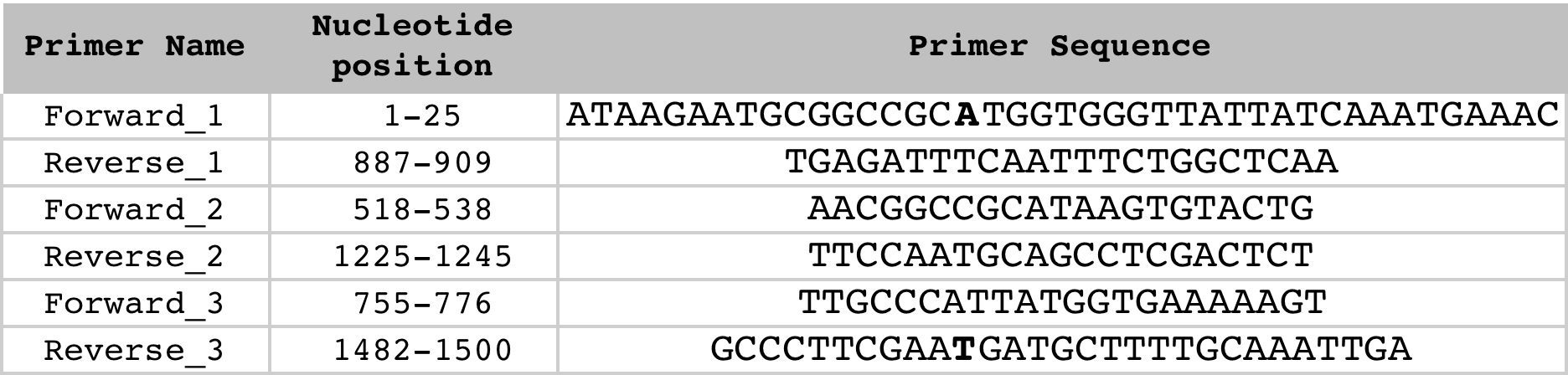
**

Three sets of primers were developed to the *Apis mellifera Kr-h1* sequence, which produced three overlapping amplicons. The position of the primers in the *AmKr-h1* mRNA sequence and the primers sequences are shown. Note that Forward_1 primer included a Not1 restriction site on the 5’ end, while Reverse_3 primer included a BstB1 restriction site on its 5’ end. The nucleotide corresponding to the start of the *Kr-h1* sequence in these primers is in bold.
