# Supplementary figures and images for: The transcription factor Krüppel homolog 1 is linked to hormone mediated social organization in bees
Source: BMC Evol Biol. 2010 Apr 30;10:120. doi: 10.1186/1471-2148-10-120 (PMC2876159; doi:10.1186/1471-2148-10-120)

**Table S2. Primer sequences for quantitative real-time PCR.**

**
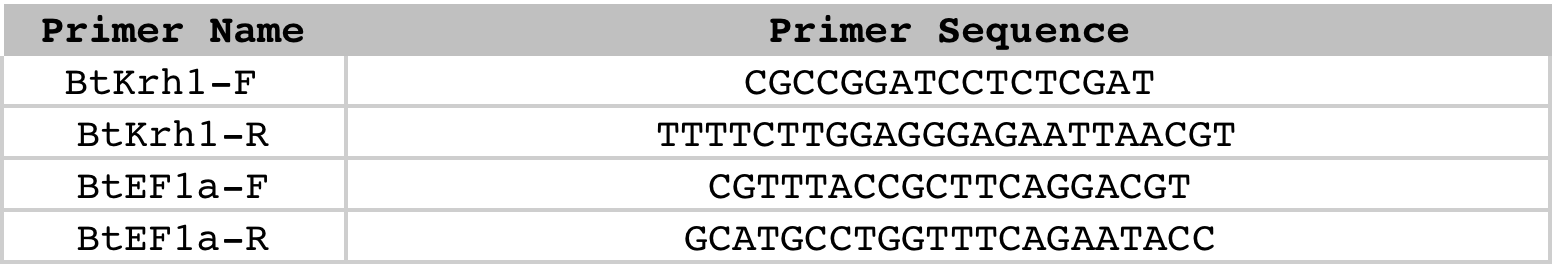
**

Supplement: Additional file 4 — Primer sequences for quantitative real-time PCR. Sequences of the B. terrestris, Kr-h1 and EF1a primers used for qRT-PCR. [file 1471-2148-10-120-S4.DOC]
