# Supplementary material for: The transcription factor Krüppel homolog 1 is linked to hormone mediated social organization in bees
Source: BMC Evol Biol. 2010 Apr 30;10:120. doi: 10.1186/1471-2148-10-120 (PMC2876159; doi:10.1186/1471-2148-10-120)
Supplement: Additional file 2 — Alignments of the insect KR-H1 orthologs. Alignment of KR-H1 orthologs from 20 insect species [file 1471-2148-10-120-S2.PDF]

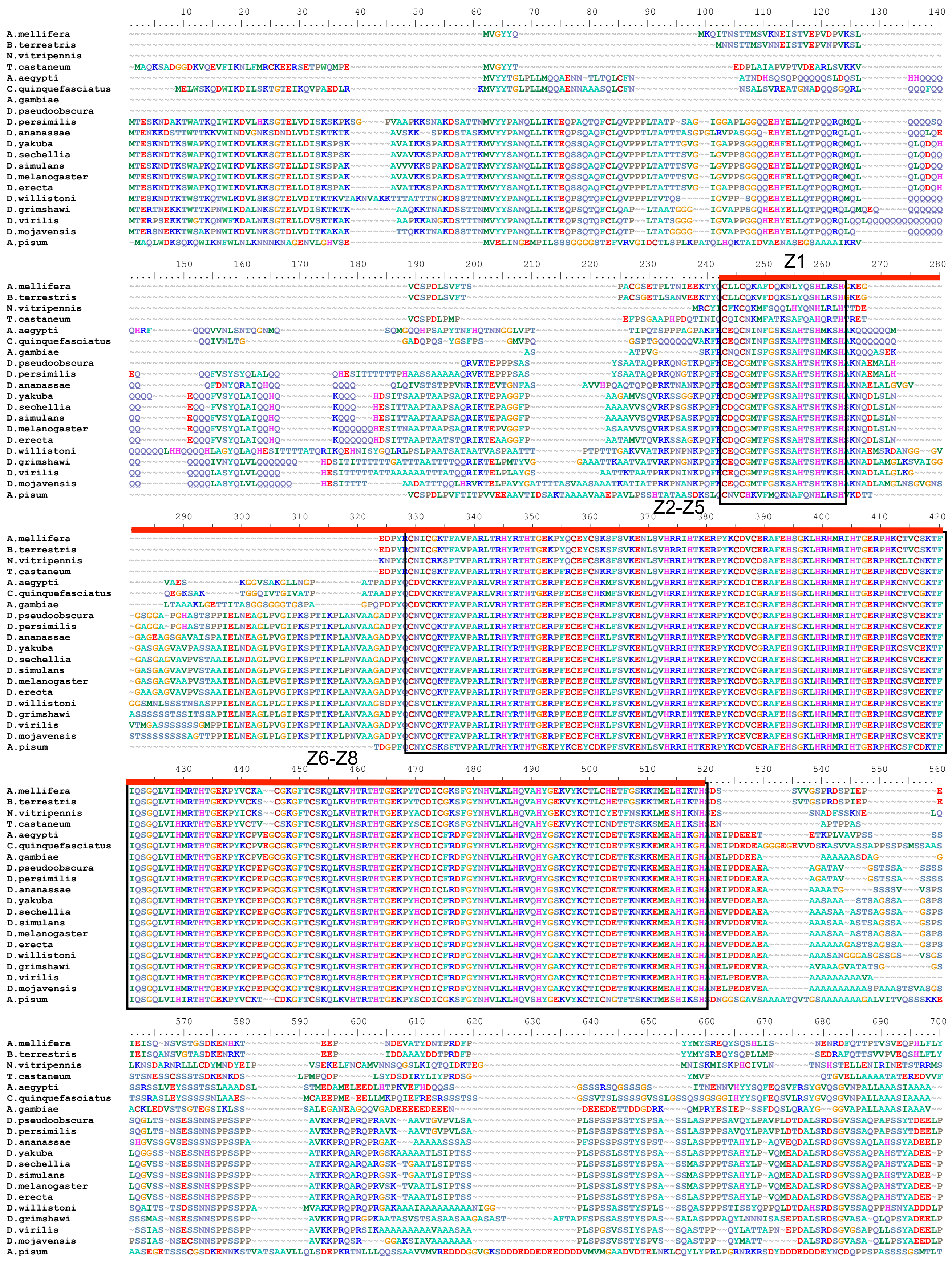
Z1
Z2-Z5
Z6-Z8
A.mellifera
B.terrestris
N.vitripennis
T.castaneum
A.aegypti
C.quinquefasciatus
A.gambiae
D.pseudoobscura
D.persimilis
D.ananassae
D.yakuba
D.sechellia
D.simulans
D.melanogaster
D.erecta
D.willistoni
D.grimshawi
D.virilis
D.mojavensis
A.pisum

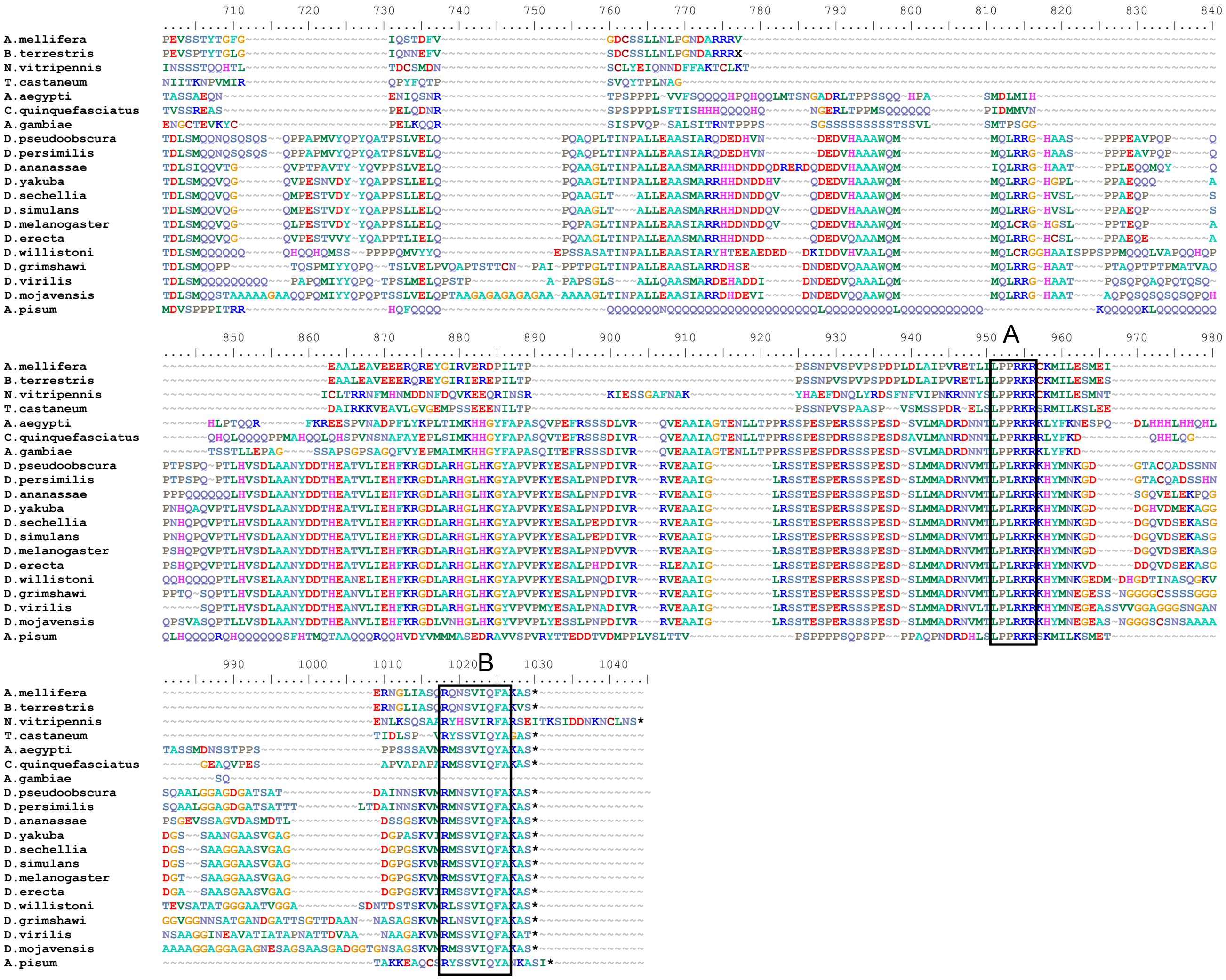
A
B
A.mellifera
B.terrestris
N.vitripennis
T.castaneum
A.aegypti
C.quinquefasciatus
A.gambiae
D.pseudoobscura
D.persimilis
D.ananassae
D.yakuba
D.sechellia
D.simulans
D.melanogaster
D.erecta
D.willistoni
D.grimshawi
D.virilis
D.mojavensis
A.pisum
